# Efficacy of Topical Administration of Corticosteroids for the Management of Dry Eye Disease: Systematic Review and Meta-Analysis

**DOI:** 10.3390/life12111932

**Published:** 2022-11-19

**Authors:** Julia Prinz, Nicola Maffulli, Matthias Fuest, Peter Walter, Andreas Bell, Filippo Migliorini

**Affiliations:** 1 RWTH University Hospital of Aachen, 52074 Aachen, Germany; 2 Department of Medicine, Surgery and Dentistry, University of Salerno, 84081 Baronissi, Italy; 3 Centre for Sports and Exercise Medicine, Barts and The London School of Medicine and Dentistry, Queen Mile End Hospital, Mary University of London, 275 Bancroft Road, London E1 4DG, UK; 4 School of Pharmacy and Bioengineering, Keele University Faculty of Medicine, Thornburrow Drive, Stoke on Trent ST4 7QB, UK; 5 Eifelklinik St. Brigida, 52152 Simmerath, Germany

**Keywords:** dry eye disease, xerophthalmus, corticosteroids, steroids

## Abstract

The efficacy of corticosteroids (CS) for dry eye disease (DED) has been investigated in the clinical setting. The present study investigated whether topical CS application improves the clinical outcome at last follow-up compared to the baseline. The present study was conducted according to the PRISMA 2020. All the randomized clinical trials (RCTs), which investigated the efficacy of corticosteroids in the management of DED, were accessed. In September 2022, the following databases were accessed: Pubmed, Web of Science, Google Scholar, and Embase. The following data were extracted at baseline and at last follow-up: Ocular Surface Disease Index (OSDI), tear breakup time test (TBUT), Schirmer I test (SIT), and corneal staining. Data from 425 patients were retrieved. A total of 69.4% (295 of 425 patients) were women. CS were effective to improve SIT (*p* = 0.02) and corneal staining (*p* = 0.003) at the last follow-up of 10.0 ± 15.3 weeks. TBUT was greater in the CS than in the control group at the last follow-up (*p* = 0.002). Concluding, topical CS administration led to an increase of SIT and a reduction of corneal staining at a mean of 10 weeks follow-up in patients with DED. Compared to a control group, topical CS administration evidenced greater values of TBUT. Altogether, a good safety profile was witnessed in DED patients receiving CS. However, different safety profiles of different CS formulations were not investigated due to a lack of quantitative data. The exact dosing frequency, duration of therapy, and favorable potency of the CS are still under investigation. Future randomized, controlled trials with larger sample sizes are warranted to provide higher-quality evidence to establish the role of CS in DED.

## 1. Introduction

Dry eye disease (DED) is very common in adults, with prevalence rates up to 50% [1]. Symptoms of DED include ocular irritation, hyperemia, dryness, and visual disorders, which can significantly affect the patients’ quality of life [1,2]. DED is diagnosed clinically by various tests, including the Schirmer I test (SIT), tear breakup time (TBUT), and corneal and conjunctival staining [3]. In addition, self-reported questionnaires, such as the Ocular Surface Disease Index (OSDI), are used [4]. 

Generally, DED results from insufficient production or excessive evaporation of tears promoting ocular surface inflammation [5]. The exact etiology of DED is still largely unknown [6]. The instability of the tear film leads to a loss of its homeostatis, which subsequently causes ocular surface inflammation and neuro-sensory abnormalities [7]. The resulting oxidative and shear stress and corneal epithelial cell damage might trigger a vicious cycle of ocular surface disruption leading to further stimulation of the inflammatory cascade by initiating both innate and adaptive immune responses [7]. An increased activation of nuclear factor kB (NF-kB) and stress-related mitogen-activated protein kinases (MAPK) trigger the production of the pro-inflammatory cytokines interleukin 1α (IL-1α), IL-1ß, IL-6, IL-8, tumor necrosis factor-α (TNF-α), and matrix metalloproteinases (MMPs) 1, 3, and 9 by corneal epithelial cells [8]. Accordingly, these cytokines have been identified in the tear film of patients with DED [9]. Antigen-presenting cells are stimulated by these cytokines, leading to an activation of CD4+ helper T cell 1, helper T cell 17, and autoantibody-secreting B cells [10]. A chronic immune response with persisting signs and symptoms of DED might be initiated [7].

Tear substitutes are the most common treatment modality for mild and moderate forms of DED [11]. However, they do not address the underlying pathogenetic factors of DED, including ocular surface inflammation [12]. Although the efficacy of anti-inflammatory agents such as lifitegrast or cyclosporine for the treatment of DED has been shown, topical corticosteroids (CS) might offer possible advantages, including the early onset of effect [12]. CS halt the inflammatory cascade by inhibiting phospholipase A2, thus impeding the conversion of phospholipids to arachidonic acid [13]. Furthermore, CS form a corticosteroid–glucose receptor complex within the cell cytosol, which downregulates angiogenesis, oxidative stress, apoptosis, and the gene expression of pro-inflammatory cytokines [13]. Moreover, CS stabilize intra- and extracellular membranes and modulate the transcription factors in mast cell nuclei [14,15]. Nevertheless, CS have been implicated with potential side effects, such as cataract formation and intraocular pressure (IOP) elevation [16]. Approximately one third of the population are moderate steroid responders, with 5% responding with IOP spikes greater than 15 mmHg after 4–6 weeks of topical CS application [17]. Therefore, topical CS are only used for a short period of time. Prednisolone and dexamethasone are classified as moderate potency formulations [18], whereas commercially available low-potency CS eye drops include loteprednol etabonate, fluorometholone, and rimexolone [19]. The low potency CS are rapidly hydrolyzed into inactive metabolites with minimal or even no effect on IOP [20]. Moreover, long-term use of topical CS is presumed to depress the immune function of the ocular surface, and thus increasing the risk of infectious, especially herpetickeratitis [21].

The efficacy of topical CS application for DED has been investigated in the clinical setting [12,15,22,23,24,25,26,27,28,29]. However, a detailed review of the literature of this evidence is lacking. Therefore, this study investigated the efficacy and feasibility of CS for DED. We evaluated the improvement from baseline to last follow-up and we performed a meta-analysis comparing CS to other commonly used pharmacological treatments. Our hypothesis is that topical CS are an efficacious treatment for DED with comparable results to other commonly used pharmacological treatments. However, long-term side effects have to be evaluated.

## 2. Materials and Methods

### 2.1. Eligibility Criteria

All of the randomized clinical trials (RCTs) which evaluated the topical steroids application for DED were accessed. Only studies published in peer reviewed journals were eligible. According to the authors’ language capabilities, articles in English, German, Italian, French, and Spanish were eligible. Only articles with level I of evidence, according to the Oxford Centre of Evidence-Based Medicine [30], were included. Reviews, opinions, letters, and editorials were not considered. Animals, in vitro, biomechanics, computational, and cadaveric studies were not eligible. Studies which combined steroids with other treatments (e.g., hyaluronic acid, antibiotics, immunomodulators) were not considered, nor were those which reported data from steroids application and from the comparator group not separately. Only studies which reported quantitative data under the outcomes of interest were considered. 

### 2.2. Search Strategy

This systematic review was conducted according to the Preferred Reporting Items for Systematic Reviews and Meta-Analyses: the 2020 PRISMA statement [31]. The PICOD algorithm was preliminarily pointed out:

- P (Population): DED;
- I (Intervention): isolated topical steroids application;
- C (Comparison): efficacy and safety;
- O (Outcomes): Ocular Surface Disease Index; tear breakup time test; Schirmer I test; corneal staining;
- D (Design): RCTs.

In September 2022 PubMed, Web of Science, Google Scholar, and Embase were accessed using the Boolean operator AND/OR. No time constrains were used for the search. The following keywords were used in combination: xerophthalmus, dry eye disease, xeropthalmia, steroids, corticosteroids cortisone, cortisol, hydrocortisone, dexamethasone, prednisolone, fluorometholone, loteprednol, medrysone, management, therapy, Ocular Surface Disease Index; Tear breakup time test; TBUT; Schirmer I test, SIT; Corneal Staining. 

### 2.3. Selection and Data Collection 

Two authors (F.M. and J.P.) independently performed the database search. All of the resulting titles were screened and, if suitable, the abstract was accessed. The full-text of the abstracts which matched the topic were accessed. If the full-text was not available or not accessible, the article was excluded. A cross reference of the bibliography of the full-text articles were also screened for inclusion. Any disagreement was discussed and settled by consensus.

### 2.4. Data Items

Two authors (F.M. and J.P.) independently performed data extraction. The following data were extracted at baseline and at last follow-up: Disease Index (OSDI) [32], tear breakup time test (TBUT) [33], Schirmer I test (SIT) [34], and corneal staining [35]. The primary outcome of interest was to investigate whether CS application will improve the clinical outcome at last follow-up compared to the baseline. The secondary outcome of interest was to compare therapy with topical CS to a control group.

### 2.5. Methodological Quality Assessment

The assessment of the methodology quality among the included studies was performed by one author (J.P.). The Review Manger software (The Nordic Cochrane Collaboration, Copenhagen, Denmark) version 5.3 was used. The focus was on the following biases: selection, detection, performance, reporting, attrition, and other biases. To assess the publication risk of bias, the funnel plot of the most commonly reported outcome was performed. 

### 2.6. Synthesis Methods

The statistical analysis was performed by one author (F.M.) following the guidelines in the Cochrane Handbook for Systematic Reviews of Interventions [9]. To assess the improvement from baseline to the last follow-up, the mean difference (MD), standard error (SE), T value, and the unpaired T-test were evaluated using the IBM SPSS software version 25. For the comparisons, the meta-analyses were conducted using the Review Manager software (The Nordic Cochrane Collaboration, Copenhagen, Denmark) version 5.3. Data were analyzed using the inverse variance and mean difference (MD) effect measure. The comparisons were performed with a fixed model effect as set up. Heterogeneity was assessed through the Higgins-I2 test. If I2 test > 50%, a random model effect was adopted. The confidence intervals (CI) were set at 95% in all comparisons. The overall effect was considered statistically significant if *p* < 0.05.

## 3. Results

### 3.1. Study Selection

**Figure 1:**
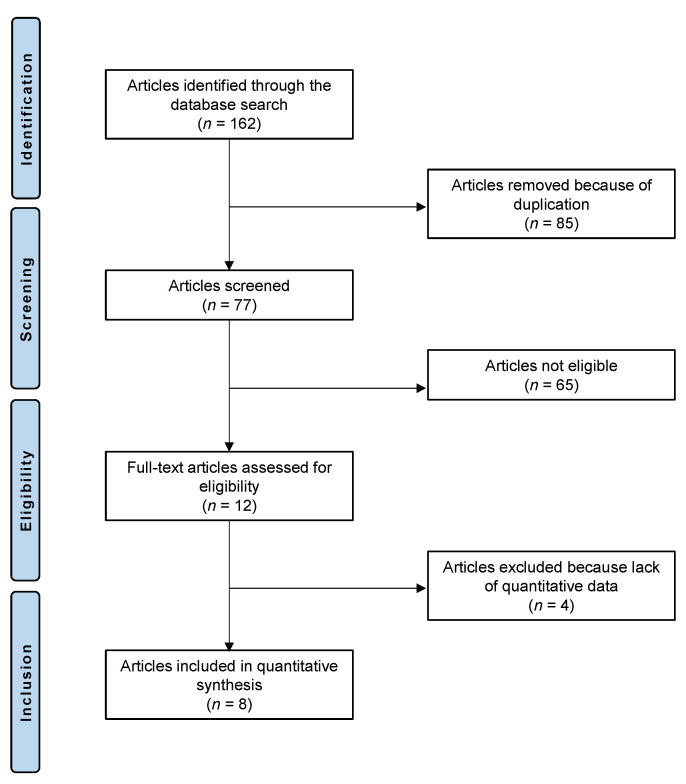
Flow chart of the literature search.

The literature search resulted in 162 RCTs which evaluated the effectiveness of acupuncture for DED. Of them, 85 were excluded because of redundancy. Another 65 articles were excluded because they did not match the eligibility criteria: type of study (*n* = 29), not focusing on the topic (*n* = 24), combining steroids with other treatments (*n* = 8), and language incompatibility (*n* = 4). Four further studies did not report quantitative data under the endpoints of interest. Finally, eight RCTs were eligible for analysis. The flow chart of the literature search is shown in Figure 1.

### 3.2. Study Risk of Bias Assessment

**Figure 2:**
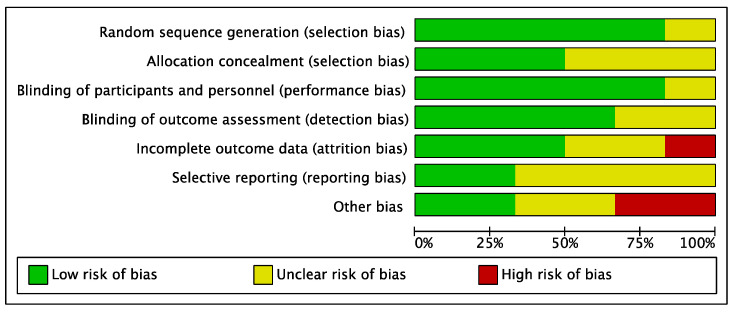
Methodological quality assessment.

Given the randomized design of the included studies, the risk of selection bias was low. Similarly, the risk of performance, selection, and detection biases were low. The overall risks of attrition and reporting biases were low to moderate (Figure 2).

### 3.3. Study Characteristics and Results of Individual Studies

Data from 425 patients were retrieved. A total of 69.4% (295 of 425 patients) were women. The mean follow-up was 10.0 ± 15.3 weeks. The mean age was 56.4 ± 4.5 years. Baseline comparability between the placebo and active treatment was found in terms of TBUT (*p* = 0.1), OSDI (*p* = 0.2), SIT (*p* = 0.09), and corneal staining (*p* = 0.1). Generalities of the included studies are reported in Table 1.

### 3.4. Efficacy of Steroids

CS were effective in reducing SIT (*p* = 0.02), corneal staining (*p* = 0.003). OSDI (*p* = 0.1) and TBUT (*p* = 0.2) did not evidence significant improvement at last follow-up. These results are shown in greater detail in Table 2. 

### 3.5. Meta-Analyses

**Figure 3:**
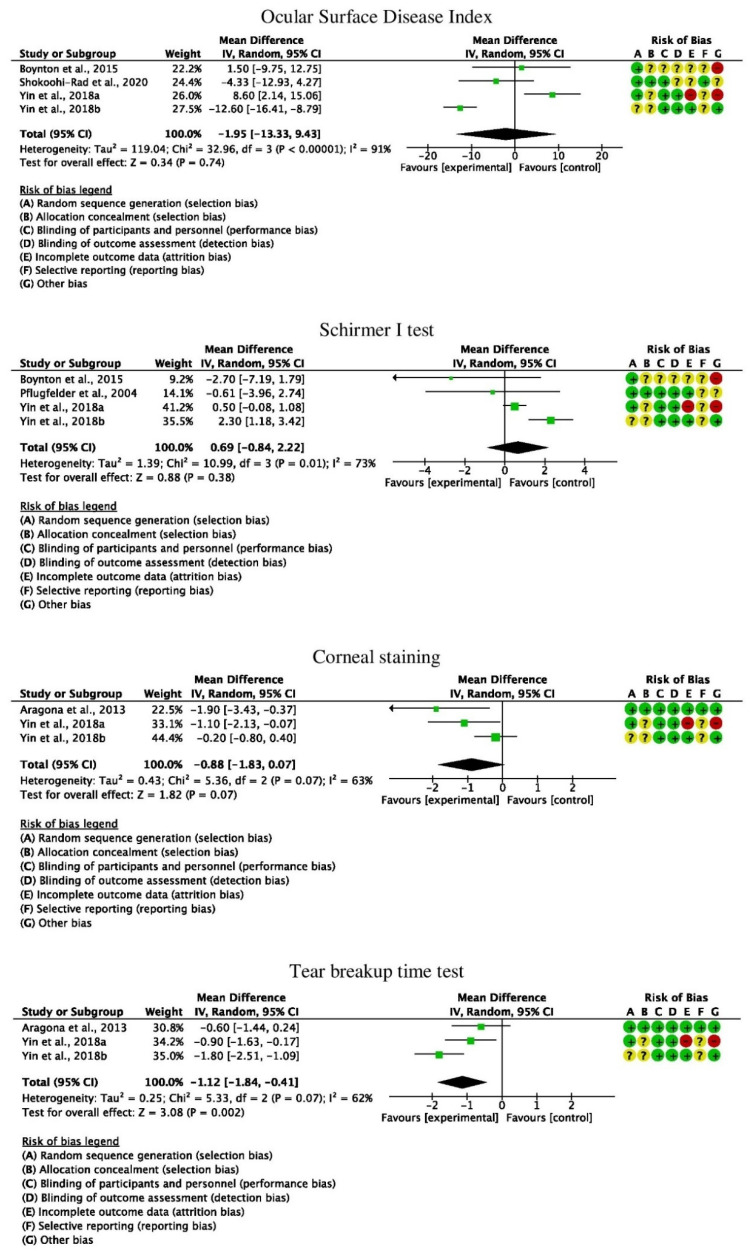
Forest plots of the meta-analyses [12,15,22,23,29,36,37,38].

The meta-analyses demonstrated no superiority of topical steroids application compared to any of the considered control groups in OSDI, SIT and corneal staining. TBUT was greater in the CS than in the control group (*p* = 0.002) (Figure 3).

## 4. Discussion

According to the main findings of the present study, topical CS administration led to an increase of SIT and a reduction of corneal staining at a mean of 10 weeks follow-up in patients with DED. Compared to a control group, topical CS administration evidenced greater values of TBUT. Altogether, a good safety profile was witnessed in DED patients receiving CS. 

To date, artificial tears are considered the first-line therapy in the management of DED [11]. However, the application of artificial tears does not address the underlying inflammatory causes of DED in patients with severe symptoms [12]. Inflammation is presumed to play a key pathogenic role for DED [39]. The efficacy of anti-inflammatory therapy, such as lifitegrast or cyclosporine, in DED has been reported previously [12]. CS are the most effective anti-inflammatory therapy for many chronic inflammatory diseases [40]. In ophthalmology, topical CS are approved for CS-responsive inflammatory conditions of the cornea, conjunctiva, and the anterior segment of the eye [29]. Previously, studies found that CS decrease the production of proinflammatory cytokines by the corneal and conjunctival epithelium [41]. CS have different propensities for side effects. Early generation CS, such as dexamethasone and prednisolone, have a strong side effect profile. In contrast, newer CS, such as loteprednol etabonate, show less risk of IOP elevation [42]. 

Kallab et al. investigated the efficacy and safety of preservative-free hydrocortisone 0.335% either for 12 days four times daily followed by 2 days twice daily (intense treatment group) or for 8 days three times daily followed by 3 days twice daily instillation (standard treatment group). They included 60 DED patients with a mean age of 51 years. OSDI significantly decreased in both treatment groups. The treatment effect remained significant 2 weeks after the treatment phase, indicating a prolonged effect of the hydrocortisone treatment. The absence of changes in IOP suggested a good safety profile of hydrocortisone [12]. The reduced likelihood for side effects was attributed to the less pronounced penetration through the ocular surface by CS formulations such as hydrocortisone [12,43]. Moreover, the authors strongly recommend the use of preservative-free CS formulations to avoid toxic side effects and to allow for a high tolerability on the ocular surface [12]. No cataract formation was witnessed. However, with a follow-up of only 4 weeks, long-term data regarding cataract formation are missing [12]. Shokoohi-Rad et al. evaluated the efficacy of a preoperative dose of betamethasone acetate 0.1% compared to placebo on DED after cataract surgery. Sixty-two patients with a mean age of 69.2 years were included. No significant effect of a preoperative betamethasone acetate instillation on postoperative DED indices, including OSDI, was reported in their study [23]. However, in the study by Shokoohi-Rad et al., only two measurements for the evaluation of DED were used: the meniscometry test and OSDI questionnaire [23]. Therefore, no conclusions can be drawn on other relevant parameters, such as the SIT or TBUT score. The authors conclude that betamethasone acetate 0.1% has no effect over placebo in DED after cataract surgery. However, this conclusion should be interpreted with caution, as the effect on the TBUT or SIT scores has not been assessed [23]. In addition, DED after cataract surgery has a different pathogenesis and is not primarily based on the inflammatory component. Rather, DED after cataract surgery has been shown to be attributable to a series of factors, including prolonged use of antibiotic–steroid eye drops, decreased mucin production from the conjunctiva following the incision, decreased corneal sensation because of the surgical incision disrupting the cornea–lacrimal gland loop, decreased TBUT due to the surface irregularity at the incision site, and the exposure to light from the operating microscope [44,45]. 

Rolando et al. included patients with DED from Sjögren’s syndrome (SS) and compared the treatment with cortisol phosphate 0.3% eye drops to cortisol phosphate 0.3% in a hyaluronic acid vehicle. No changes in IOP were witnessed. Furthermore, no cases of cataract formation were observed. The formula with the hyaluronic acid vehicle proved to be more effective, with a significant improvement of corneal and conjunctival staining at 7 days compared to baseline in comparison with the cortisol phosphate 0.3% group. However, it should be considered that the cortisol phosphate 0.3% eye drops contained preservatives with low levels of direct toxicity on histology of the tissues [46]. Therefore, the preservatives might be a confounding factor and hypothetically contribute to the difference witnessed between the two groups [22]. Nevertheless, the authors argue that the combination of cortisol phosphate associated with hyaluronic acid creates a compound between the steroid and the polymer, which is a hydrophilic mucoadhesive drug. In this drug, cortisol and hyaluronic acid are linked through hydrogen bonds, which enable the drug to bind the epithelial corneal mucin, leading to a long permanence of the CS on the ocular surface [22]. Pinto-Fraga et al. assessed the efficacy of topical fluorometholone 0.1% compared to polyvinyl alcohol in 40 DED patients. After a follow-up of 3 weeks, greater improvements of corneal and conjunctival staining, hyperemia, and TBUT were witnessed in the fluorometholone compared to the polyvinyl alcohol group. No significant IOP changes occurred [15]. In addition, an increase in the best corrected visual acuity was witnessed in the fluorometholone group, which can be attributed to the positive correlation between corneal epithelial damage and visual acuity [47]. Interestingly, the authors found the 0.1% fluorometholone therapy to prevent an ocular surface worsening in DED patients exposed to desiccating stress. Therefore, Pinto-Fraga et al. suggest this treatment to be occasionally administered to patients who are anticipating adverse environmental conditions, including air-conditioned vehicles, etc. Especially, the treatment could be used to avoid exacerbation periods rather than in the long-term [15]. Aragona et al. evaluated the efficacy of clobetasone butyrate eyedrops in patients with DED associated with SS. Forty patients were included in the RCT. A significant improvement of corneal and conjunctival staining at last follow-up compared to baseline occurred in patients treated with topical clobetasone butyrate. No severe adverse events, including no cases of IOP elevation or cataract formation, were witnessed [36]. Yin et al. investigated the efficacy of loteprednol etabonate 0.5% ophthalmic suspension in two groups of 21 patients each with DED, with or without association to graft-versus-host-disease (GVHD), respectively. Similar to the inflammation cascade described above, DED in GVHD is mediated by T cells and cytokines [48]. The authors concluded a less favorable efficacy of loteprednol etabonate 0.5% in patients with ocular GVHD [38]. This lower efficacy in the GVHD group is attributed to different baseline characteristics between the two groups, possibly including cellular and molecular markers of inflammation. In addition, chronic ocular GVHD is characterized by lacrimal tissue destruction and fibrosis, which might not be reversible by topical CS treatment at an advanced stage [38]. Similarly, Boynton et al. analyzed the efficacy of loteprednol etabonate 0.5% compared with cyclosporine A in the management of DED associated with GVHD. In both groups, similar changes in OSDI, SIT, corneal staining, and TBUT occurred without relevant complications, such as IOP elevation [37]. However, as ocular hypertension is a common complication in patients with ocular GVHD, the long-term application of topical CS has to be considered with caution [49]. In a study by Pflugfelder et al., 66 patients were randomized to receive either topical loteprednol etabonate 0.5% or only the vehicle of loteprednol etabonate ophthalmic suspension. Loteprednol etabonate led to significant higher improvement in inferior tarsal and nasal bulbar conjunctival hyperemia at 2-week follow-up and in nasal bulbar hyperemia at 4-week follow-up than the vehicle [29]. The authors suggest that patients with greater degrees of ocular surface inflammation show a greater likelihood for a clinical response to loteprednol. Moreover, no toxic effects nor cases of IOP elevation due to the therapy with topical loteprednol were evidenced [29]. Interestingly, both loteprednol and its vehicle contained a preservative (benzalkonium chloride), which is known to cause corneal epithelial toxicity [50]. However, a clinical improvement in conjunctival hyperemia was witnessed at 2 weeks, while the improvement was less impressive at 4 weeks [29].

Three further RCTs investigating the efficacy of CS in DED are registered on https://clinicaltrials.gov (accessed on 4 September 2022). A RCT (NCT04711642) is planned to be conducted by the Fundación Oftalmológica Los Andes in Chile to evaluate the efficacy of a topical CS combined with antibiotic (tobramycin 0.3% and dexamethasone acetate 0.1%) on DED prior to and after cataract surgery. MMP9 is used as a parameter to diagnose ocular surface dysfunction. Recruitment of 100 patients has not started yet. Another registered RCT (NCT04498468) sponsored by the Johns Hopkins University, Maryland, USA, compares the efficacy of a CS intracanalicular insert releasing dexamethasone for up to 30 days to a sham comparator. Recruitment of an estimated patient enrolment of 85 patients has started. 

Between studies, both the CS groups and the control groups were variable, and may impact the reliability of the results of the present meta-analysis. The efficacy of CS was compared to placebo in the studies by Aragona et al. [36] and Shokoohi-Rad et al. [23], to topical polyvinyl alcohol in the study by Pinto-Fraga [15], or to cyclosporine A in the study by Boynton et al. [37]. Yin et al. used artificial tears as a comparator [38]. Kallab et al. compared the efficacy of two different treatment protocols (standard and intense protocol) of hydrocortisone 0.335% [12]. Rolando et al. compared the efficacy of cortisol phosphate 0.3% eye drops to cortisol phosphate 0.3% in a hyaluronic acid vehicle as a control group [22]. Given the lack of quantitative data available for inclusion in the literature, no further subgroup investigations regarding different CS formulations were possible in the present meta-analysis. Furthermore, no subgroup investigations concerning different control groups was possible. Active treatments, such as cyclosporine A, and inert substances, such as placebo, were included as control groups, which might limit the reliability of the results of the present study. Moreover, no subgroup investigations in terms of formulations containing preservative versus preservative-free eye drops were possible. Additionally, the heterogeneous length of the follow-up might limit the reliability of our results. Moreover, it remains unclear whether the parameters investigated in this study, especially corneal staining, were evaluated identically, given the different investigators and implementation options. Given the lack of quantitative data, the different steroids (e.g., hydrocortisone, betamethasone, fluorometholone) and related concentration and administration protocols were not analyzed separately. Future long-term investigations are required to overcome these limitations and compare different CS with related concentration and administration protocols in a clinical setting. Therefore, the most indicated therapeutical algorithm should be identified and defined by future larger cohort level I studies. Given these limitations, results from the present study must be interpreted with caution. The current literature would benefit from high-quality clinical trials on a large scale. Future high-quality studies are needed to confirm the role of CS in patients with DED.

## 5. Conclusions

Topical CS administration led to an increase of SIT and a reduction of corneal staining at a mean of 10 weeks follow-up in patients with DED. Compared to a control group, topical CS administration evidenced greater values of TBUT. Altogether, a good safety profile was witnessed in DED patients receiving CS. However, different safety profiles of different CS formulations were not investigated due to a lack of quantitative data. The exact dosing frequency, duration of therapy, and favorable potency of the CS are still under investigation. Future randomized, controlled trials with larger sample sizes are warranted to provide higher-quality evidence to establish the role of CS in DED.

## Figures and Tables

**Table 1:** Generalities and patient baseline of the included studies.

Author, et al., Year	Journal	Follow-Up (Weeks)	Patients (*n*)	Treatment	Dosages of CS	Duration of CS Instillation	Mean Age	Women (%)	Outcomes of CS Group
Aragona et al., 2013 [36]	*Eur J Ophthalmol*	4.3	20	Control group			60.1	95.0	**TBUT**: significantly improved from baseline to last FU; significantly improved compared to control group **Corneal staining**: significantly improved from baseline to last FU
			20	CS (Clobetasone butyrate)	0.1%	30 days	58.8	95.0	
Boynton et al., 2015 [37]	*Cornea*	52.1	38	CS (Loteprednol)	0.5%	13 months	54.1	47.4	**OSDI**: no significant differences **SIT**: no significant differences
			37	Control group			51.2	48.6	
Kallab et al., 2019 [12]	*Adv Ther*	4.0	30	CS (Hydrocortisone)			51.0	66.7	**OSDI**: significantly decreased at the last follow-up compared to baseline (in both CS treatment groups)
			30	CS (Hydrocortisone)	0.335%	14 days vs. 11 days	51.0	66.7	
Pflugfelder et al., 2004 [29]	*Am J Ophthalmol*	4.0	32	CS (Loteprednol)		28 days	57.6	62.5	**SIT**: no significant differences
			34	Control group			56.2	88.2	
Pinto-Fraga et al., 2016 [15]	*Ophthalmology*	3.0	21	CS (Fluorometholone)		22 days	59.0	81.0	**TBUT**: significantly improved compared to control group **Corneal staining**: significantly improved compared to control group
			19	Control group	0.1%		60.3	89.5	
Rolando et al., 2017 [22]	*J Ocul Pharmacol Ther*	4.0	20	CS (Cortisol)	0.3%	28 days	60.1	95.0	**Corneal staining**: CS + hyaluronic acid group significantly improved from baseline to last FU and significantly improved compared to CS only group
			20	CS (Cortisol) + hyaluronic acid			58.8	95.0	
Shokoohi-Rad et al., 2020 [23]	*Indian J Ophthalmol*	4.3	28	CS (Betamethasone)	0.1%	31 days	66.0	50.0	No significant differences between the groups
			34	Control group			64.6	52.9	
Yin et al., 2018 [38]	*Am J Ophthalmol*	4.0	10	CS (Loteprednol)			55.6	70.0	**OSDI**: significantly decreased at the last follow-up compared to baseline only in non-GVHD patients
			11	Control group	0.5%		61.4	81.8	
			12	CS (Loteprednol)			55.8	50.0	
			9	Control group			50.6	55.5	

CS: corticosteroids; TBUT: tear breakup time test; last FU: last follow-up.

**Table 2:** Analysis of the improvement of TBUT (s), OSDI (points), SIT (mm), and corneal staining (points) from baseline to the last follow-up.

Endpoint	Baseline	Last FU	MD	SE	95% CI	*p*
TBUT	3.0 ± 0.7	3.1 ± 0.4	0.1	0.08	−0.0590 to 0.2590	0.2
OSDI	40.6 ± 23.6	33.0 ± 20.3	−7.6	3.11	−13.7388 to 0.4612	0.1
SIT	8.0 ± 3.7	6.1 ± 2.5	−1.9	0.45	−2.7806 to −1.0194	0.02
Corneal staining	4.5 ± 2.2	3.1 ± 1.7	−1.4	0.28	−1.9483 to −0.8517	0.003

FU: follow-up; MD: mean difference; SE: standard error; CI: confidence interval; OSDI: Ocular Surface Disease Index; TBUT: tear breakup time test; SIT: Schirmer I test.

## Data Availability

Not applicable.
